# Obesity and Outcome of Crohn’s Associated Perianal Fistula Surgery: A Case-Control Study

**DOI:** 10.14740/gr698e

**Published:** 2015-12-31

**Authors:** Ashish Manne, Ali S. Khan, Talha A. Malik

**Affiliations:** a Department of Medicine-Gastroenterology, University of Alabama at Birmingham, Birmingham, AL, USA; b Department of Epidemiology, University of Alabama at Birmingham, Birmingham, AL, USA

**Keywords:** Obesity, Crohn’s disease, Perianal fistula

## Abstract

**Background:**

Evidence suggests that obesity (body mass index (BMI) > 30 kg/m^2^) adversely affects several outcomes in Crohn’s disease (CD). CD-associated perianal fistula (CDPF) represents a debilitating phenotype with a clinical course that may be affected by obesity. We hypothesized that obese CD patients would be more likely to have poor outcomes following CDPF surgery.

**Methods:**

We designed a case-control study of CD patients who underwent surgery for CDPF between 2000 and 2013 with documented pre-operative BMI and post-operative outcome. Cases and controls were defined based on the outcome of CDPF surgery. Poor outcomes were designated as cases.

**Results:**

Of the 317 patients diagnosed with CDPF, 73 patients underwent 120 surgeries for CDPF. Eighty-nine (74%) of the surgeries comprised fistulotomy with or without Seton placement, whereas 31 (26%) were mucosal flap procedures. Twenty-five (21%) cases and 95 (79%) controls were identified. Unadjusted odds ratio (OR) for the association between obesity and outcome demonstrated a trend towards a poor surgical outcome among obese patients that did not reach statistical significance (OR: 1.86; 95% confidence interval (CI): 0.58 - 5.98; P = 0.295). Multivariable logistic regression analysis demonstrated an even stronger trend towards a poor outcome among obese CD patients, albeit without reaching statistical significance (OR: 2.83; 95% CI: 0.64 - 12.49; P = 0.169).

**Conclusion:**

In patients undergoing Seton placement, fistulotomy or mucosal flap procedure for CDPF, there is a trend towards poor outcomes in the obese; however, as this trend did not reach statistical significance, this association should be examined further.

## Introduction

Crohn’s disease (CD) is a heterogeneous chronic inflammatory condition, primarily of the intestinal tract that affects 750,000 people in the US. It is characterized by transmural inflammation and can involve any part of the gastrointestinal tract. Despite well-characterized genotypic and phenotypic characteristics of affected patients, CD responds variably and unpredictably to treatment [1-3].

Perianal fistula is a common complication of CD. According to Schwartz et al, perianal fistulas occur in as many as 20% of Crohn’s patients with recurring episodes after initial management in 33% [4]. Perianal fistulas can be classified based on the anatomy and location [5-7] or severity of the disease (anal disease activity index [8], perianal disease activity index (PDAI) [9], and Pikarsky’s perianal CD activity index [10]). Though examination under anesthesia, endoscopy, MRI, endoanal ultrasound, fistulography and CT are used in diagnosis and staging these fistulas, it is advocated to combine two or more of these available modes for an accurate picture [11].

Medical management with tumor necrosis factor (TNF) blockers is very effective in treating CD-associated perianal fistula (CDPF) [12]. Combining TNF blockers with surgery has shown to be more effective than medical management alone or surgery alone especially in those who have complications such as abscess and incontinence[13, 14].

Adipose tissue is an active endocrine organ that is made up of elements of connective tissue as well as cells represented by pre-adipocytes and adipocytes that can be prominent mediators of inflammation in the human body [15-18]. Body mass index (BMI), the most commonly used clinical measure of the degree of adipose tissue in the body, is calculated by dividing weight (in kilograms) by the height (in meters) squared and a BMI of ≥ 30 kg/m^2^ is categorized as representing “obesity” [15]. In the past few decades, the prevalence of obesity throughout the US and the world has markedly increased [19]. This trend in obesity is reflected in patients with CD as well [20, 21]. Study of the relationship between obesity and objective outcomes in CD is especially important because of credible molecular evidence that links adipose tissue physiology to intestinal inflammation. However, it is unclear whether this link translates into a causal or clinically meaningful and consistent association between obesity and CD outcome such as surgical outcome in perianal fistula surgery.

Several studies over the years have indeed demonstrated that obesity is an adverse factor in CD patients affecting its progression and management (both medical and surgical) [22-25]. A 2013 study at our institution demonstrated a general association between obesity and poor outcome in surgeries done on patients with CD. Surgeries examined in our previous study included intestinal resections, diverting ileostomies and perianal fistula procedures [26]. While in clinical practice, obese Crohn’s patients are considered to be poor candidates for any type of surgery and not just surgery involving the perineum, some studies have been equivocal in this regard. In the current study, we sought to understand the influence of obesity on the subset of Crohn’s patients who underwent perianal fistula surgeries.

## Materials and Methods

### Study design, patient population, and selection criteria

Following approval by the University of Alabama’s Office of the Institutional Review Board, we began a retrospective unmatched case-control study of men and women 19 years and older with CD who underwent surgery for a complication related to CDPF at University of Alabama at Birmingham Hospital (UAB) between 2000 and 2013. These patients were retrospectively identified using a health system billing search engine. Data on pre-operative BMI, demographics and post-surgical outcome in the patients had to be available for them to be included into the study. Patients were excluded if they had any current history of cancer, were receiving chemotherapy or had missing data. Women who underwent perianal surgery for rectovaginal fistula and persons who underwent surgery for colovesical or rectovesical fistula were also excluded.

### Data

In both cases and controls, information obtained from review of patient charts included: 1) demographic data (age, ethnicity, gender, and the BMI); 2) clinical variables (smoking history, duration of disease and clinical disease activity at the time of surgery); 3) medication history (use of steroids, traditional immune modulators and biological agents); and 4) type of surgery.

Cases were represented by unsuccessful or poor surgical outcomes. Specifically, unsuccessful or poor surgical outcome was defined as any post-operative complication elicited during the post-operative recovery period, or during the first post-operative scheduled or unscheduled visit that required a non-standard intervention. Complications were characterized by post-operative wound infections, delayed wound healing or prolonged recovery, wound dehiscence, development of an abscess or death. Any perianal fistula surgery, which was not followed by any of these complications was considered a control.

Obesity was measured using BMI. BMI was calculated by dividing a person’s weight in kg by height in meters squared. Based on the BMI, patients were divided into two groups. Obese patients were those with a BMI of greater than 30 kg/m^2^. The rest had a BMI between 18.5 and 29.9 kg/m^2^, and they were classified as non-obese. Pre-operative height and weight were used to calculate BMI. Self-reported height and weight were not used to avoid recall bias.

CD clinical activity was documented for each patient in accordance with the American College of Gastroenterology (ACG) practice guidelines. Patients were divided into two categories, those in remission or mild to moderate disease, and those with moderate to severe disease. History of corticosteroid use was defined as more than 1 week of conventional oral or parenteral steroids, or more than a month of rectal or topical steroids or more than 3 months of oral budesonide within the past 1 year. Any history of oral, parenteral, rectal, topical steroids including use of oral budesonide within the 1 month prior to surgery was considered recent steroid use.

Immune modulator use was considered positive if a patient was on azathioprine (AZA), 6-mercaptopurine (6-MP), methotrexate (MTX) or any biological agent prior to or at the time of surgery and was on the particular agent at the first post-operative follow-up appointment. Smokers were defined as those who were smoking at the time of surgery.

### Statistical analysis

Demographic characteristics and clinical information were compared between cases and controls using Chi-square test or Fisher exact test for categorical variables and independent *t*-test for continuous variables. However, if evidence showed that the data did not follow a normal distribution, Wilcoxon rank sum test, a non-parametric analog of the *t* test, was used instead. Unadjusted odds ratios (ORs) and 95% confidence intervals (CIs) were estimated for the association between BMI as a continuous as well as categorical (obese vs. non-obese) variable and outcome of interest (surgical outcome) by logistic regression. We selected age, gender, and ethnicity, duration of disease and duration of observation as covariates in adjusted models. Finally, multivariable logistic regression analyses adjusted for age, gender, and ethnicity, duration of disease and duration of observation were performed. Statistical tests were two-sided with a 5% significance level (i.e., α = 0.05). Statistical software (Statistical Analysis Software (SAS), version 9.2; SAS Institute, Inc., Cary, NC) was used to perform all statistical analyses.

## Results

At UAB, between 2000 and 2013, of the 317 patients with perianal fistulas, 73 patients underwent 120 perianal fistula surgeries. Eighty-nine (74%) of the surgeries comprised fistulotomy with or without Seton placement, whereas 31 (26%) were mucosal flap procedures. Of these 120 surgeries, 25 (21%) were cases, whereas 95 (79%) represented controls. There was a difference in median BMI between cases (approximately 23 kg/m^2^) and controls (25 kg/m^2^) (P = 0.813). The proportion of obese patients was higher among cases compared to controls (20% of cases vs. 12% of the controls) (P = 0.282). Remarkable difference was also seen in the presence of extra intestinal manifestations (28% of cases vs. 6% of controls) (P = 0.005). A comparison of other characteristics of cases and controls appears in Table 1. Specifically, there was no significant difference between cases and controls in regard to ACG-based clinical disease activity index.

**Table 1:** Comparison of Other Characteristics of Cases and Controls

Characteristic	Cases (N = 25)	Controls (N = 95)	P-value
BMI, median (range) (n = 118)	22.9 (12.3 - 38)	24.9 (17.3 - 68.7)	0.813
BMI categorical (n = 118)			
Obese	5 (20%)	11 (12%)	0.282
Overweight	4 (16%)	28 (30%)	
Standard weight	16 (50%)	54 58%)	
Age at surgery, median (range)	34 (19 - 55)	38 (19 - 78)	0.049
Age at diagnosis, median (range)	22 (8 - 53)	29 (2 - 72)	0.054
Year of surgery, median (range)	2008 (2002 - 2012)	2008 (2003 - 2013)	0.619
DOD, median (range)	8 (1 - 28)	5 (1 - 30)	0.683
Ethnicity, African American	11 (44%)	30 (32%)	0.244
Past CDPF surgery	18 (72%)	43 (45%)	0.024
Sex, women	15 (60%)	54 (57%)	0.776
Smoking (n = 119)	5 (20%)	22 (23%)	0.718
Steroid use	4 (16%)	16 (17%)	1.00 (Fisher)
AZA/6-MP use	11 (44%)	37 (39%)	0.646
Biologic use	13 (52%)	47 (49%)	0.822
Methotrexate use	1 (4%)	14 (15%)	0.191 (Fisher)
EIM	7 (28%)	6 (6%)	0.005

The unadjusted OR for the association between obesity and outcome demonstrated a trend towards a poor surgical outcome among obese patients that did not reach statistical significance (OR: 1.86; 95% CI: 0.58 - 5.98; P = 0.295). Multivariable logistic regression analysis adjusted for covariates that had an effect size of > 10% on the association between obesity and surgical outcome (age at surgery, age at diagnosis, ethnicity, past CDPF surgery, extra-intestinal manifestations) demonstrated an even stronger trend towards a poor outcome among obese CD patients, albeit without reaching statistical significance (OR: 2.83; 95% CI: 0.64 - 12.49; P = 0.169).

## Discussion

In this case-control study, we attempted to study the effect of obesity on the sub-set of Crohn’s patients who specifically underwent perianal fistula surgery (excluding women who underwent perianal surgery for rectovaginal fistula and persons who underwent surgery for colovesical or rectovesical fistula). We drew inspiration from our previous study where we demonstrated that obesity was indeed a risk factor for poor overall surgical outcome in CD [26].

CD has gained a lot of attention in the past few years due to a dramatic rise in its incidence and prevalence. Owing to poor awareness regarding this disease, many patients are being diagnosed after complications such as fistula, abscess, perforation or bowel obstruction which often require surgery among various other treatments. As we learn more about this disease, its pathophysiology and its progression, the focus has now shifted to identifying factors which affect its prognosis.

Obesity is an established risk factor for poor outcome in CD. Obese patients have increased severity of disease and also have a poor outcome after appropriate standard management when compared to non-obese patients. In 2013, we tried to understand the effect of obesity on the overall outcomes of patients who undergo any surgery for CD (and its related complications) with 90 eligible candidates. We noted that among obese patients who underwent a Crohn’s related surgery, rate of unsuccessful outcome was 64% compared to a rate of unsuccessful outcome of 41% among the non-obese [26].

In this present case-control study, we attempted to study the effect of obesity on the sub-set of Crohn’s patients who underwent surgery for perianal fistula (excluding rectovaginal, colovesical, and rectovesical fistulas).

Small sample size was an important limitation of this study. Additionally, we were unable to ensure documentation of post-operative outcome independent of clinical status. Controls in our study were identified at the end of observation (cumulative sampling) which may have led to selection bias. To decrease information bias, we relied almost solely on medical record documentation verified by a second data abstractor for both components of BMI (height and weight) as well as for data on other covariates. Given the similarities in the usage of steroids, AZA/6MP and biologics in the two groups, confounding by indication was not an issue however. We did note that MTX usage was more likely to occur in controls when compared to cases. Another potential limitation was that we were not able to accurately classify perianal fistulas based on Parks classification and therefore did not include this in the variables. Another important limitation was our inability to limit the actual perianal surgical procedures to one type and had to lump fistulotomies, Seton placements and flap procedures together, again largely on account of a relatively small sample. Also, there were other important differences between cases and controls in regard to potential risk factors. However, our hypothesis was limited to looking at the effect of obesity and therefore the findings and their interpretation was limited to this particular association.

Consequently, this current study shows a definite trend of poor outcome of perianal fistula surgery in obese patients with CD. However, this association needs further examination in larger prospective studies.

## Abbreviations

CD: Crohn’s disease
BMI: body mass index
CDPF: CD-associated perianal fistula
TNF-alpha: tumor necrosis factor alpha
AZA: azathioprine
6-MP: 6-mercaptopurine
MTX: methotrexate
SAS: Statistical Analysis Software
